# Electrical stimulation to prevent recurring pressure ulcers in individuals with a spinal cord injury compared to usual care: the Spinal Cord Injury PREssure VOLTage (SCI PREVOLT) study protocol

**DOI:** 10.1186/s13063-022-06088-0

**Published:** 2022-02-16

**Authors:** Boas J. Wijker, Sonja de Groot, Johanna M. van Dongen, Femke van Nassau, Jacinthe J. E. Adriaansen, Wendy J. Achterberg-Warmer, Johan R. Anema, Andries T. Riedstra, Maurits W. van Tulder, Thomas W. J. Janssen

**Affiliations:** 1 Department of Human Movement Sciences, Faculty of Behavioural and Movement Sciences, Vrije Universiteit Amsterdam, Amsterdam, The Netherlands; 2 Amsterdam Rehabilitation Research Center | Reade, Amsterdam, The Netherlands; 3 Department of Health Sciences, Faculty of Science, Vrije Universiteit Amsterdam, Amsterdam, The Netherlands; 4 Department of Public and Occupational Health, Amsterdam UMC, Vrije Universiteit Amsterdam, Amsterdam Public Health Research Institute, Amsterdam, The Netherlands; 5 Reade, Center for Rehabilitation & Rheumatology, Amsterdam, The Netherlands; 6 Paraplegia Organization | Dwarslaesie Organisatie Nederland (DON), Nijkerk, The Netherlands; 7 Faculty of Behavioural and Movement Sciences, Vrije Universiteit Amsterdam, Amsterdam, The Netherlands

**Keywords:** Spinal cord injuries, Electrical stimulation, Pressure ulcer, Prevention and control, Secondary prevention, Cost-benefit analysis, Process evaluation, Randomized controlled trial

## Abstract

**Background:**

Pressure ulcers (PUs) on the buttocks are among the most common secondary complications in individuals with chronic spinal cord injury (SCI). PUs can result from sitting for extended periods, disuse atrophy, increased sitting pressure and reduced circulation. Compared with usual care, activation of paralysed muscles using electrical stimulation (ES) has been shown to markedly increase paralysed muscle mass, improve circulation of skin and muscle and improve sitting pressure distribution. ES might therefore be a useful method to reduce PU incidence.

**Methods:**

A multicentre randomized controlled trial (SCI PREVOLT) will be conducted with an economic and process evaluation alongside. One hundred participants with a SCI in the chronic phase and a minimal incidence of 1 PU in the last 5 years will be recruited from rehabilitation centres across the Netherlands. Participants will be stratified by centre and age and randomized to the intervention or control group. The intervention group will use ES at least 1 h/day during at least 4 times a week for 1 year next to usual care. The control group will only receive usual care. The primary outcome is the incidence of PUs, measured by a blinded person assessing the presence or absence of a PU on the buttocks on a photo made by the participant or his/her caregiver. The incidence of a PU will be evaluated every 2 weeks. Secondary outcomes include interface pressure distribution, blood flow in the profunda femoris artery, muscle thickness of the hamstrings and gluteal muscles and questionnaires about different dimensions of life, e.g. participation and quality of life. Secondary outcomes will be measured at baseline and 3, 6, 9 and 12 months after randomization.

**Discussion:**

This study will assess if electrical stimulation is a (cost-)effective method to prevent PUs and reduce the risk factors of getting PUs. If ES is effective and cost-effective compared with usual care, ES could be implemented in daily treatment of individuals with a SCI.

**Trial registration:**

Netherlands Trials Register NTR NL9469. Registered on 26 May 2021.

**Supplementary Information:**

The online version contains supplementary material available at 10.1186/s13063-022-06088-0.

## Administrative information

**Table:** 

Title {1}	Electrical stimulation to prevent recurring pressure ulcers in individuals with a spinal cord injury compared to usual care; the Spinal Cord Injury PREssure VOLTage (SCI PREVOLT) study protocol
Trial registration {2a and 2b}.	NTR ID: NL9469, Registered 26 May 2021
Protocol version {3}	Version 2.61 date 21-10-2021
Funding {4}	ZonMw Efficiency Studies Program, Grant no: 853001111
Author details {5a}	1. Boas J. Wijker PT, (PhD candidate) 2. Sonja de Groot, PhD 3. Johanna M. van Dongen, PhD 4. Femke van Nassau, PhD 5. Jacinthe Adriaansen, MD & PhD 6. Wendy J. Achterberg – Warmer, MD 7. Johannes R. Anema, PhD 8. Andries Riedstra, MSc 9. Maurits W. van Tulder, PhD 10. Thomas W.J. Janssen, PhD
Name and contact information for the trial sponsor {5b}	Name: ZonMw Telephone number: 070 349 51 11 E-mail: info@zonmw.nl
Role of sponsor {5c}	ZonMw has no control or authority over further development of the study design, collection, management, analysis, interpretation of the data, writing the report or where to submit the report during the research.

## Introduction

### Background and rationale {6a}

Pressure ulcers (PUs), a common secondary complication in individuals with spinal cord injury (SCI), are characterized by damage to the skin and/or underlying tissue due to unrelieved pressure or pressure in combination with shear forces [1]. PUs develop following a prolonged period of compression and usually occur over bony prominences [2–6]. This compression of the tissue between a bony prominence, the skin and a surface, causes in turn the occlusion of capillaries leading to tissue ischemia and necrosis [7–9]. In severe cases, PUs can even be life-threatening [10–12].

Individuals with SCI are at an increased risk of developing PUs due to factors, such as reduced mobility, inability to adequately release pressure, atrophy of the paralysed muscles, a disturbed leg muscle pump function, reduced microcirculation and impaired sympathetic function [13]. Up to 85% of all individuals with SCI develop a pressure ulcer (PU) once or more in their life [6, 14–16]. A Dutch multicentre study found the occurrence of PUs during inpatient rehabilitation to be 37–39% [17]. Results of Adriaansen et al. [18] showed that the prevalence of a PU was 42%, 41% and 29% among individuals with SCI, 1, 2 and 5 years respectively after discharge. Two other studies found the overall PU incidence was still 29% after 10 years for individuals living with SCI [19, 20]. Many individuals with SCI suffer from recurrent PUs (> 1 every 3 years), with 40–80% of those who develop a PU having at least one recurrence [12]. Verschueren et al. [17] showed that having had a PU during the acute rehabilitation phase was the strongest predictor for a recurrence.

Usual PU preventive care starts during inpatient rehabilitation on a specialized SCI unit and follows the multidisciplinary guideline “Decubitus preventie en – Behandeling” [21], the Dutch translation of the international guidelines of the National and European Pressure Ulcer Advisory Panel [22]. This guideline consists of advice on choosing an adequate wheelchair, anti-PU cushion, lifestyle and nutrition, pressure-relieving movements, training how to perform adequate transfers, visually skin condition self-checks and psychosocial therapy, if needed. However, these methods do not meet the demands necessary for adequate management and prevention of PUs [1].

#### Relevance

After being diagnosed with a PU, treatment can either be conservative or surgical (debridement) and reconstructive and very often consists of mandatory prolonged bed rest to release pressure on the wound(s). Even though bed rest alleviates pressure on the wound, it also has some severe negative consequences, such as decreased mobility and independence and reduced physical condition, health-related quality of life and level of participation. On top of that, PUs are associated with high societal costs due to increased health care utilization and work absenteeism [2, 3, 23]. Consequently, preventing the recurrence of PUs will likely lead to a reduction of the burden to individuals with SCI and society.

#### Electrical stimulation

A method to potentially prevent the recurrence of PU is activating the paralysed muscles using electrical stimulation (ES). ES has been found to markedly improve PU risk factors by improving paralysed muscle mass and local circulation of skin and muscle [6, 24–30]. However, randomized controlled trials (RCTs) to confirm the effect of ES on reduction of PU recurrence are lacking and the cost-effectiveness of ES in individuals with SCI is unknown. Even though ES is not a new intervention, it has not yet been implemented widely in clinical SCI rehabilitation practice, largely due to the lack of well-controlled studies.

#### Existing evidence of the effectiveness of electrical stimulation

Preliminary studies found that intermittently activating the gluteal muscles during 30 min using surface ES acutely reduced interface pressures [31]. Studies also showed positive results in a reduction of interface pressure and blood flow restoration when performing ES on the gluteal and hamstring muscles. This was performed for a longer stimulation period (3 h) in a more daily-life situation in which participants wore a lycra garment with built-in electrodes and used a portable stimulator [32–35]. More recently, Barton et al. [36] performed a study involving nine individuals with a chronic SCI, who performed 12 weeks of a self-administered and self-chosen daily gluteal and hamstring stimulation, which showed that ES results in transduced structural and functional changes in the femoral artery supplying the active skeletal muscle, an improved sitting pressure distribution, an increased muscle mass and a moderately improved cutaneous microvascular function at the gluteal site.

In summary, ES seems promising in reducing potential risk factors of developing recurrent PU or a PU itself. However, a study evaluating the effect of ES as a preventive method for PUs is missing in the literature. Therefore, an RCT is needed to assess the effectiveness and cost-effectiveness of electrical stimulation plus usual care on the occurrence of PUs compared with only usual care.

### Objectives {7}

#### Primary objective

1. To investigate whether daily ES of gluteal and hamstring muscles combined with usual care is more effective in reducing recurrences of PUs than only usual care in individuals with chronic SCI, who had minimally 1 PU within the last 5 years.

#### Secondary objective(s)

1. To investigate whether daily electrical stimulation of gluteal and hamstring muscles combined with usual care is more effective than usual care only in improving:

- Factors related to PU risk: interface pressure distribution, local circulation and muscle size;
- Mobility, participation and quality of life;

In individuals with chronic SCI, who had minimally 1 PU within the last 5 years.

2. To determine the cost-effectiveness of ES compared with usual care alone.
3. To investigate facilitators and barriers for the implementation and sustainability of daily ES of gluteal and hamstring muscles combined with usual care.
4. To evaluate the usability and user-friendliness of the ES system.

### Trial design {8}

A prospective multicentre RCT will be conducted with an economic and process evaluation alongside. One hundred participants with a chronic SCI will be randomly assigned to either an intervention or control group. It is a superiority trial that aims to evaluate whether daily ES (intervention group) is more effective than usual care (control group) on the prevention of pressure ulcers.

## Methods: participants, interventions and outcomes

### Study setting {9}

The aim is to carry out this study in all eight Dutch rehabilitation centres with a specialized SCI unit: Reade centre for rehabilitation and rheumatology, location Overtoom, located in Amsterdam, Adelante located in Hoensbroek, Heliomare located in Wijk aan Zee, de Hoogstraat rehabilitation located in Utrecht, Roessingh located in Enschede, Rijndam located at Rotterdam, Sint Maartenskliniek located in Nijmegen and UMCG, location Beatrixoord, located in Haren.

### Eligibility criteria {10}

#### Inclusion criteria

To be eligible for this study, an individual must meet all of the following criteria:

- Individuals with either a complete (AIS A) or incomplete (AIS B, C and D) chronic SCI^1^
- Age 18 years and older
- Intact gluteal and hamstring muscles^2^
- A minimal incidence of 1 PU or more of category II-IV in the sacral or ischial tuberosity’s region within the last 5 years^3^
- Able to lie in a prone position for at least 10 min (safely for the neck, comfortable, not compromising breathing and taking possible contractures into account)

#### Exclusion criteria

Exclusion criteria are:

- Current PUs in the gluteal or sacral area
- Flaccid paralysis (areflexia)
- A history of severe autonomic dysreflexia
- Insufficient mastery of the Dutch language (speaking and reading)
- Severe cognitive or communicative disorders
- Intolerance to or contra-indication for ES (cancer, pregnant)
- Recent or current participation in an ES-induced exercise program or study (up to 6 months prior to this study)
- Severe psychiatric illness or disorders (to the discretion of the treating rehabilitation physician)

### Recruitment {15}

Participant recruitment will occur between Start in May 2021 for approximately 1 year. Recruitment consists of approaching a potential participant in two different ways, i.e. active and inactive recruitment.

For the active recruitment strategy, rehabilitation physicians, Physician Assistants (PA), wound nurse and interns will distribute flyers and the information letter among individuals who may qualify for the study. Both will be distributed during a discharge interview, an outpatient appointment or at the patient’s follow-up interview 1 year after discharge. If a potential participant immediately expresses his/her interest in participating in the study, the practitioner will ask for permission to pass on his/her contact details (name and telephone number) directly to the local research assistant. The practitioner will then inform the patient that this local appointed and trained research assistant will contact them by telephone to make an appointment for the physical screening to check for eligibility. So, in the case of active recruitment, there will be no telephone pre-screening because the practitioners will only hand out flyers to potential participants who they regard will (at least) partially meet the inclusion and exclusion criteria.

For the inactive recruitment strategy, flyers will be distributed within the eight Dutch SCI rehabilitation centres and within the network of the Dutch SCI patient organization (Dwarslaesie Organisatie Nederland (DON)). Within the DON, the flyer will be distributed via social media and the flyer will be published in their magazine. When someone is interested in participating, he/she can contact the executive or principal investigator directly by email or telephone, using the contact details provided in the flyer. The executive or principal investigator will then perform a pre-screening interview via telephone. A telephone pre-screening interview is chosen in order to not unnecessarily burden potential participants with a visit to the rehabilitation centre. During this pre-screening, a brief explanation is given about the research. Subsequently, permission is asked to ask questions about the health of the potential participant in order to assess whether someone is suitable for study inclusion. If someone meets these written inclusion and exclusion criteria, permission is again requested to pass on the contact details to a local research assistant. The local research assistant from the nearest rehabilitation centre will make an appointment with the potential participant for the physical screening and will send the information letter via email prior to the screening (at least 5 working days due to the reflection time).

### Who will take informed consent? {26a}

The physical screening is carried out by the local investigator or trained research assistants and consists of reassessment of the inclusion and exclusion criteria, an extensive oral explanation of the research study and the opportunity to ask questions. If someone agrees to participate, the informed consent will be signed in duplicate. After this, the physical inclusion or exclusion criteria will also be assessed, namely the contraction of the hamstring and gluteal musculature. If these inclusion criteria have also been met, the participant will be included in the study. After the baseline questionnaire is fully completed, the participant will be randomized to the intervention or control group. For the informed consent, see Appendix 2.

## Assignment of interventions: allocation

### Implementation {16c}

#### Sequence generation {16a}

Randomization will be done by the research assistant of Reade. Participants will be randomly assigned to either the intervention or control group in a 1:1 ratio with a web-based randomization program (studyrandomizer.com). A permuted block algorithm will be used with a variable block size with a minimum of 4, a maximum of 8 and an increment of 2. Randomization will be stratified for rehabilitation centre and for two age groups; i.e. 18–70 years and ≥70 years. Randomization list will only be available to the local researcher of the rehabilitation centre, the executive investigator and principal investigator. The research assistant of Reade is also the one who completes the Case Report Form (CRF) and keeps everyone posted on how many participants are enrolled to the study.

#### Concealment mechanism {16b}

Participants will be randomized using the program Study Randomizer (studyrandomizer.com), which is a web-based randomization service. Allocation concealment will be ensured, as the service will not release the randomization code until the patient has been recruited into the trial, which takes place after all baseline questionnaires and measurements have been completed.

#### Additional consent provisions for collection and use of participant data and biological specimens {26b}

Not applicable, no biological samples will be collected.

## Interventions

Explanation for the choice of comparators {6b}

Participants will be randomly assigned to one of the following two groups: (1) Control group that will only receive usual care or (2) Intervention group that will receive daily ES and usual care.

### Intervention description {11a}

#### Usual care

The control group will only receive usual care according to the national multidisciplinary guideline “Pressure ulcer prevention and treatment” (Decubitus preventie en – Behandeling) [21], i.e. the Dutch translation of the international guidelines of the National and European Pressure Ulcer Advisory Panel [22]. These guidelines consist of multidisciplinary advice on choosing an adequate wheelchair, anti-pressure ulcer cushion, (air-) matrasses, healthy lifestyle, nutrition, transfer training, learn to perform pressure-relief movements and psychosocial therapy (if needed).

#### Intervention group

The intervention group will receive usual care plus ES. ES will be applied using a 4-channel portable stimulator (EMP 4 Eco+; Wuxi Jiajian Medical Instrument Co., Ltd; China) connected with surface electrodes, either self-adhesive electrodes or a custom-made lycra garment with built-in surface electrodes (Berkelbike BV, Sint-Michielsgestel, NL). The researchers decide who receives a garment or surface electrodes while looking at the following criteria; the participant’s choice, functionality, independence, the presence of wounds on the back and the presence of a stoma. ES will be given for at least 60 min bi-phasically with a 6 s:18 s activation-rest cycle, 50 Hz (frequency), a 300–400-μs pulse duration and the output current amplitude (mA) will be determined per participant. The current amplitude resulting in the best tetanic contraction without creating discomfort or excessive muscle contractions will be determined for each participant individually during the baseline measurement by the ES expert or research assistant. This amplitude will be used as starting point for the home stimulation, participants will be instructed to increase the mA if no activation or only twitches are visible. The activation within the activation-rest cycle consists of 2 s ramp-up, 3 s full activation and 1 s ramp-down followed by 18 s rest. The 6 s:18 s activation-rest cycle is based on the 1:6 cycle which has been proven to be effective and seems more effective than a cycle with less rest [34, 37]. The 1:6 cycle is multiplied by 3 to create a longer contraction and rest period, which might lead to a lower risk for long-term muscle fatigue. The researchers present it as a 6 s:18 s activation-rest cycle, since the ramp-up and down are also seen as activation.

### Criteria for discontinuing or modifying allocated interventions {11b}

When one of the following adverse events or inconveniences occurs during ES, the intervention needs to be stopped temporarily:

- Red, raised or itchy skin [38–40]
- Muscle pain [38, 40]
- Increase in nerve pain
- Feeling uncomfortable [38, 40]
- Orthostatic hypotension: dizziness, light-headedness, blurred vision, palpitation or shortness of breath [38, 41]
- Autonomic dysreflexia, dysfunction or dysregulation
- Pain induced by spasms

The adverse events mentioned above are temporary and usually disappear once the stimulation has stopped. If problems continue to arise during stimulation, the intervention will be stopped.

### Strategies to improve adherence to interventions {11c}

Not applicable, this trial will not use strategies to improve adherence to the intervention.

### Relevant concomitant care permitted or prohibited during the trial {11d}

Not applicable, this trial does not have concomitant care permitted or prohibited.

#### Use of co-intervention

During the intervention period, participants will be asked not to participate in any other ES-induced exercise program, or to participate in any other study that may interfere with the current study.

### Provisions for post-trial care {30}

The materials provided to the intervention group such as the adhesive surface electrodes of the ES garments may be kept for personal use. The stimulator EMP4 ECO+ needs to be handed in at the last physical measurement. If the intervention shows to have a positive effect, the control group will receive advice and guidance about ES after the study is completed.

### Outcomes {12}

PU incidence, the primary outcome parameter, will be monitored every 2 weeks throughout the 12-month study period. Secondary outcome parameters (i.e. potential PU risk factors, mobility limitations, health-related quality of life, participation and usability of the ES system) will be evaluated at baseline (T0) and after 3 (T1), 6 (T2) and 12 months (T4). Cost data will be gathered at baseline and every 3 months so also at 9 months (T3). Process evaluation questionnaires will be administrated every 3 months and interviews will be performed at 3 and 12 months. Table 1 provides an overview of all assessment moments and when the measurements take place.

**Table 1:** Schedule of assessments and overall measurements

Time (months)	0	1	2	3	4	5	6	7	8	9	10	11	12
**Time points**	**T0**			**T1**			**T2**						**T3**
**Primary outcomes**													
New PU*		X	X	X	X	X	X	X	X	X	X	X	X
Picture		X	X	X	X	X	X	X	X	X	X	X	X
**Secondary outcomes*: PU risk factors***													
Blood flow	X			X			X						X
Muscle thickness	X			X			X						X
Interface pressure distribution	X			X			X						X
**Secondary outcomes*: Questionnaires***													
PASPID	X			X			X						X
WHO-QoL-5	X			X			X						X
USER-participation	X			X			X						X
SIP-68	X			X			X						X
HADS	X			X			X						X
PSQI	X			X			X						X
FSS	X			X			X						X
Self-care capacity	X			X			X						X
***Usability and user-friendliness of the ES system***													
Self-administrated questionnaire				X			X			X			X
Diary		X	X	X	X	X	X	X	X	X	X	X	X
***Cost analysis***													
EQ-5D-5L	X			X			X			X			X
Online cost questionnaire	X			X			X			X			X
***Process evaluation***													
Questions of the RE-AIM model				X									X
Interviews				X									X
Focus group													X

*Every 2 weeks (if the answer is yes, also data are collected about the number of days bedrest AND absence of work)

#### Baseline characteristics

At baseline, the following determinants, which can have an effect on developing PUs and are potential confounders, are also collected: age, sex, cause of SCI, level of SCI, Asia Impairment Scale, time since injury, rehabilitation centre, number of PUs in the past, locations of PUs in the past, PU surgery in the past (yes/no and how often), type of surgery, comorbidities (heart and vascular, stomach liver, intestinal or kidney disease, bladder, urinary or respiratory problems, diabetes, history with cancer, musculoskeletal or other neurological disorders) and spasms (yes/no and if yes, which muscles).

#### Primary outcome

##### Pressure ulcer incidence

PU incidence in the sacral or ischial tuberosity’s region (in total and for each severity category according to the EPUAP [22]) will be recorded throughout the 12-month study period by the participant or his/her caregiver. Every 2 weeks, each participant will receive an email or notification with the question of whether a new PU has been identified or whether an existing PU is still present. If the answer is ‘yes’ then the following steps need to be taken:

- A digital photo of the wound will be made by the participant or his/her caregiver according to standardized instructions and will be sent to a nurse practitioner specialized in wound care. All pictures will be analysed by the same well-trained and experienced nurse practitioner. The location of the wound and the severity (category 1–4) will be classified according to the EPUAP [22]. If there is a wound visible in the photo the wound is also evaluated with the TIME model, a method for evaluating local disruptive factors [21].
- Change in risk score will be evaluated with a modified CBO list, derived from the main elements of the Norton, Waterlow, Braden scale and 2 Dutch lists, i.e. PrePURSE and CBO list [1].
- (Possible) Cause of PU needs to be described by the participant.
- Healing tendency will be calculated by the experienced nurse practitioner by dividing the starting content (depth wound) or surface (superficial wound) by the number of days to complete wound healing. The outcome will be the average number of surface area reduction per day. This can only be done in case of a wound severity category 3 or 4 [42].

Each participant will be asked to take a standard photo of the sacral and ischial tuberosities regions every month. This picture should be taken regardless of a pressure ulcer is present.

### Secondary outcome measures

The PU risk factors will be measured at baseline, 3, 6 and 12 months. Questionnaires will be sent out at baseline, 3, 6 and 12 months.

#### PU risk factors

Prior to the measurement, the participant will be asked not to eat for ≥ 6 h, smoke or consume tobacco ≥ 6 h, consume alcohol or food/drinks that contain caffeine or are rich in polyphenols ≥ 12 h and to avoid exercise ≥ 24 h [43].

#### Local circulation

The participant will be transferred from the wheelchair to a bed and placed in a supine position. After a 20-min resting period [36] the local circulation will be determined by measuring the Blood Flow with Color Doppler in the profunda femoris artery (deep femoral artery). The artery will first be measured in Brightness mode (b-mode) transversal for localization just proximal to the femoral bifurcation. Then the probe will be rotated till longitudinal and slide distally just after the femoral bifurcation where the profunda femoris artery lays most superficial for data collection. The Lumify L12-4 (Philips, USA) is a 12-4 MHz linear array transducer which will be attached to a Samsung Galaxy tab S7+ (Model SM-T970). Color Doppler will be obtained using the Fast Flow function of the software and with the lowest possible insonation angle (always < 60°) [43]. Data analysis will be done with a custom-designed script in Matlab which is made in collaboration with Philips and will include custom edge-detection, wall tracking and colour flow analysis.

#### Muscle thickness

Muscle thickness of the hamstrings (biceps femoris) and the gluteal muscles will also be measured using the Philips Lumify L12-4 ultrasound system.

The muscle thickness of the gluteal muscles will be measured separately due to the different anatomical position origin and insertion wise. So there is made a separate scanning protocol to capture the gluteus maximus and one which captures the medius and minimus in one picture. For both measurements, the participant will lay in a lateral position. Both measurements will start with palpating the trochanter major as a landmark. From there the probe will be placed longitudinally towards the most superficial part of the trochanter major, so with the caudal side of the probe (the side towards the trochanter) rotated 10–15° more posterior. From there, the probe slides a couple of centimetres more cranially so the fossa of the ilium will be visible [44].

- For the gluteus maximus, the probe will be moved more posterior to the thickest part of the gluteus maximus. This is located on the inner upper quadrant of more medial from the posterior superior iliac spina and the piriformis line [45]. There the probe will be rotated 35–45° till perpendicular to the muscle in the longitudinal axis.
- For the gluteus medius and minimus, the probe will be moved more cranial and medial towards the groin area. There the fossa of the ilium will be used as a bony landmark which is visible as a small dent.

The biceps femoris (long head) will be measured with the participant lying in a partial prone/side position. The measurement will be performed in B-mode and starts from the origin of the hamstring (ischial tuberosity) with the probe transversal. From there the probe will be rotated, till in the longitudinal axis and slide downwards and slightly laterally to follow the biceps femoris long head [44].

In both cases, a 3-s clip will be made, while making a fan shape movement to visualize the thickest part of the muscle. When the thickest part is visible, 1 picture will be made in B-mode and saved for later offline analysis using Image J. The whole process repeats till 3 pictures are collected. The first step of the analysis will be to draw lines on the muscle contours to highlight the superficial and deep aponeurosis. Step two is drawing straight lines between both aponeuroses; one in the middle of the muscle and either one or two lines relatively more to the right and left (how much depends on the visibility of the muscle). The distance between the lines between both aponeuroses determines the muscle thickness at different parts of the muscle. The average muscle thickness will be calculated by adding the measured distances and dividing them by the number of lines drawn [46–48].

#### Interface pressure distribution

Interface pressure distribution will be measured using a Boditrak PRO2 (Vista Medical), a 46.5 × 46.5 cm soft flexible mat with 1024 sensors (32×32), each measuring a maximum of 260 mmHg at a 60-Hz frequency. Each participant will be measured while sitting still in his/her own wheelchair with an adaptation time of 5 min followed by a 5-min measurement. Using custom Matlab routines, the mean ischial tuberosities pressure and the pressure gradient (indicative of shear force) will be calculated. The prominent pressure points visible in the measurement, with the highest pressure value are defined as the ischial tuberosities (left and right IT). A selection of the 3 × 3 sensors surrounding the highest values will be used to calculate the mean IT pressure. The pressure gradient will be calculated by subtracting the average of the 16 surrounding sensor values from the IT pressure.

#### Self-reported outcome measures

##### Mobility and activity limitation

*Mobility and activity limitation* will be measured with the Dutch Physical Activities Scale for Individuals with Physical Disabilities (PASIPD) [49, 50].

##### Health-related quality of life

*Health-related quality of life* will be measured with the World Health Organization Quality of Life-5 questionnaire (WHO-QoL-5) [51]. For the economic evaluation, health-related quality of life will also be expressed in terms of Quality Adjusted Life Years (QALYs). For this purpose, the EQ-5D-5L will be administered. The participants’ EQ-5D-5L health states will be converted into utility scores ranging from 0 (“death”) to 1 (“optimal health”) using the Dutch tariff, and QALYs will be estimated using linear interpolation between measurement points.

##### Participation

*Participation* will be measured with two scales; the Utrecht Scale for Evaluation of Rehabilitation- Participation (USER-participation) [52] and the 68-item Sickness Impact profile (SIP-68) [53]. Both scales will be used because the SIP-68 measures influence of illness and/or health complaints on daily functioning while the USER-participation also measures objective data like how many hours spent in a certain activity.

##### Anxiety and depression

*Anxiety and depression* will be evaluated with the Hospital Anxiety and Depression Scale (HADS) [54].

##### Sleep quality

*Sleep quality* will be evaluated with the Pittsburgh Sleep Quality Index (PSQI) [55].

##### Fatigue

*Fatigue* will be evaluated with the Fatigue Severity Scale (FSS) [56].

##### Self-care capacity

*Self-care capacity* will be evaluated with a combined questionnaire and exist of questions in the field of health and SCI. First part includes 18 of the 22 performance-related questions of Bloemen-Vrencken et al. which will have 4 answering options which are scored as follows: never (0), sometimes (1), often (2) and always (3). Total score ranges from 0 (never performing any health behaviour) up to 54 (always performing all health behaviours) [57].

The second part consists of 15 knowledge-related questions earlier used in a Dutch study but not yet described in literature. The questions have 5 answering options and are scored as follows: certainly not true (0), not true (1), no opinion (2), true (3) and certainly true (4). Total score ranges from 0 (no knowledge related to SCI) and 60 (has all the knowledge and information about SCI). A total health behaviour score will be calculated by summing up the scores of each item.

##### Usability and user-friendliness

*Usability and user-friendliness* will be evaluated with a self-administrated questionnaire and a diary. The questionnaire will exist of open or Likert-scale questions. The Likert-scale questions will have 6 answering options: totally disagree (1), disagree (2), no opinion (3), agree (4), totally agree (5) and not applicable (0). For the diary, the participants or caregivers will be asked to manually track usage of the ES system 2 times per month. The stimulator usage will also be directly recorded by the stimulator software, storing information on program use, frequency, duration of use and intensity of stimulation for thirty days. However, participants are expected to prefer manual tracking due to the complexity of the stimulator.

##### Cost data

Costs will be assessed from a societal and a healthcare perspective. From the societal perspective, intervention, other healthcare, unpaid productivity, informal care, presenteeism and absenteeism costs will be included, whereas only costs accruing to the formal Dutch health sector will be included for the healthcare perspective. Resource use data will be collected using cost questionnaires administrated at baseline 3, 6, 9 and 12 months. Intervention costs will be micro-costed. The use of other healthcare services (i.e. primary healthcare, secondary healthcare, medication) will be valued using Dutch standard costs and prices derived from an online pricelist of pharmaceutical products (www.medicijnkosten.nl). Informal care (i.e. care by family and friends) and unpaid productivity losses (i.e. productivity losses related to the fact that participants might not be able to perform unpaid activities, such as volunteer work) will be valued using a recommended Dutch shadow price. Presenteeism and absenteeism will be assessed using a modified version of the IMTA Productivity Cost Questionnaire (iPCQ). Presenteeism and absenteeism will be valued using gender-specific price weights and the friction cost approach will be used for valuing absenteeism [58].

##### Process evaluation

Parallel to the RCT, a mixed methods process evaluation will be conducted using the RE-AIM model [59]. The aims of the process evaluation are (1) to evaluate recruitment, reach and implementation of the daily ES, and participants’, care takers’ and key stakeholders’ experiences of taking part in daily ES to explain program outcomes, including unintended outcomes; (2) to evaluate the views of rehabilitation specialist on the usage of ES as a rehabilitation method; (3) to investigate facilitators and barriers for the adoption, implementation and maintenance of daily ES of gluteal and hamstring muscles combined with usual care; (4) to explore dose-response association between the degree of adherence and effectiveness; (5) to provide recommendations for improvement of the intervention beyond the research setting. The mixed-method design of this process evaluation will include interviews with; the participant, caregivers and specialist. Focus groups with a rehabilitation specialist, researchers, nurse practitioners coordinators and managers, field notes and a question included in the ES questionnaire and diary [60, 61]. Interviews will be held at T1 and after T3, focus group at T3, while questionnaire and diary data will be collected throughout the trial. For further details, see Appendix 1.

### Participant timeline {13}

**Fig. 1:**
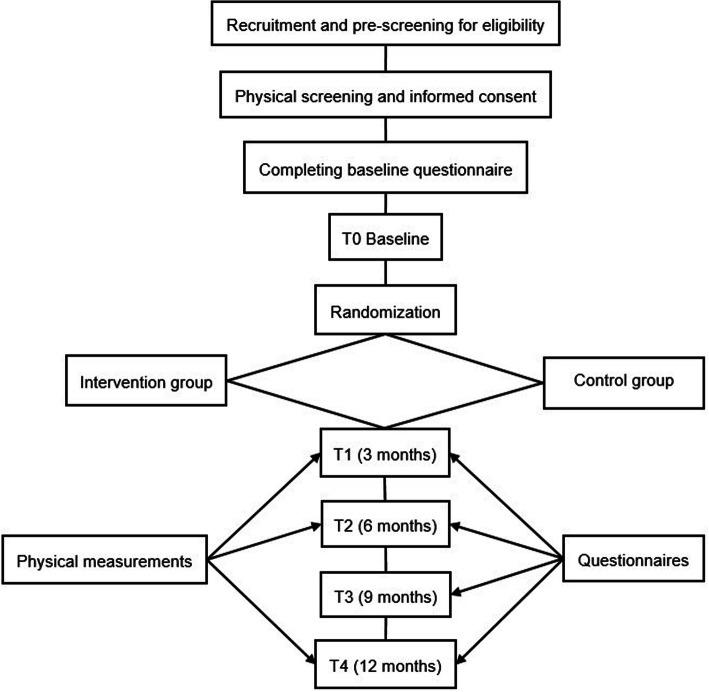
Study design flow chart

Figure 1 shows the study design flow chart with the participant timeline.

### Sample size {14}

The sample size for this study is based on the anticipated difference in the selected primary outcome, i.e. PU incidence. To detect a clinically relevant difference in incidence of PUs of 25% (35% vs. 10%) with alpha = 0.05 and power = 90%, 45 participants in each group are needed. Taking into account a dropout rate of 10%, we need to recruit a total of 100 participants (i.e. 50 participants per group).

## Assignment of interventions: blinding

### Who will be blinded {17a}

The wound care nurse, who will analyse the digital photos of the sacral or ischial tuberosity’s region (primary outcome), will be blinded during the whole trial. The assessors who will measure the PU risk factors (secondary outcome) will be blinded during the physical measurements by asking participants to not reveal the group they are in. The process evaluation will be conducted by the researchers (FvN) and (BW). Due to the qualitative nature of the research methods, both researchers will not be blinded during the study. The local research assistants in the rehabilitation centres will not be blinded because of their direct contact with the participants while providing the equipment and instructions and the need to be available for questions about ES. Except for the PI (TJ), all other researchers (SG, MvT and HvD) will be blinded during the trial and analyses.

### Procedure for unblinding if needed {17b}

Breaking the randomization code will only occur in the event of unexpected serious adverse events associated with the intervention. In that case, this will be noted on the CRF form, including the reason why, by the principal investigator (TJ) and/or executive researcher (BW).

## Data collection and management

### Plans for assessment and collection of outcomes {18a}

Not applicable, this trial does not have biological specimens.

### Plans to promote participant retention and complete follow-up {18b}

Every 2 weeks, participants will get an invitation to fill in some questions regarding the PU incidence and the intervention group will also be asked to complete the diary. When opening the online questionnaire, an overview is visible showing which questionnaire has already been completed and which questionnaire has not yet been completed. When a questionnaire is not fully completed or submitted a week after the invitation, the local researcher of Reade will contact the participant.

### Data management {19}

All data collected during this trial will be coded and de-identified and, if possible, anonymized/pseudonymized to protect the privacy of our participants and to allow open access publication of our data after the completion of the trial on the basis of the FAIR principles (69). During the course of the trial, data storage will be controlled by the PI (TJ) and the executive researcher (BW). All physically collected data (e.g. screenings forms and informed consent) will be stored in a secure locker with restricted access. All other collected data will be stored directly on Research Drive (SURF, Utrecht, The Netherlands).

The paper-based screening forms and informed consent forms will be filed by the local research assistant of the participating centres. He/she will scan all forms and upload them to Research Drive, after which he/she will collect some additional information to complete the Case Report Form (CRF). Data will be stored and managed in accordance with the quality standards of the VU Amsterdam, the Netherlands. All data collections, changes and interpretations will be documented in a codebook, which will be saved on Research Drive as well. This will be done for review and reuse purposes. Physical measurement data will be processed using Matlab and ImageJ, after which the data will be imported to SPSS. Statistical analyses will be done in SPSS, STATA and R. Throughout the data cleaning process, every step will be documented and in the scripts/syntaxes. When students will get involved in data collection/cleaning process, a short protocol will be made. A more detailed Data Management Plan (DMP) is written for this trial and available Appendix 3.

### Confidentiality {27}

The outcome data will be separately stored from the participant-identifying information and participants will be coded with a unique study ID. The personal information will only be accessible for the local researcher of the rehabilitation centre, the principal investigator and executive investigator. Rights of access will be strictly regulated by the executive researcher (BW).

### Plans for collection, laboratory evaluation and storage of biological specimens for genetic or molecular analysis in this trial/future use {33}

Not applicable, no samples collected.

### Statistical methods for primary and secondary outcomes {20a}, {20c}

Descriptive statistics will be used to describe the participants’ baseline characteristics: age, gender, level of SCI, ASIA score, time since injury, number of previous PUs. Potential confounders will be identified by adding them to the regression equations and checking whether they change the regression coefficient by more than 10% or based on the literature. If there are any clinically relevant differences between groups at baseline, we will include these potential confounders in the analysis.

#### Effect analysis

An intention-to-treat analysis will be performed using a logistic mixed model with having had a PU during 12-month follow-up (yes/no) as a dependent variable and as independent variables the study group variable (intervention or usual care group) and any potential confounding variables. Two levels will be identified; i.e. rehabilitation centre and patient. The necessity of using a random intercept and/or a random slope will be assessed using the -2 log likelihood test. If both turn out not to be necessary, a regular logistic regression analysis will be performed. Also, as maximum likelihood estimation cannot be used to deal with missing data in these analyses, missing data will be multiple imputed using Multivariate Imputation by Chained Equations (MICE) and pooled estimates will be calculated using Rubin’s rules [57].

An intention-to-treat analysis will also be performed for the secondary outcomes using a linear mixed model for continuous outcomes (e.g. quality of life). Three levels will be identified: i.e. rehabilitation centre, patient and time. Within these models, the secondary outcomes will serve as the dependent variable, a study group variable (intervention or usual care group), interaction terms for study group and time and all confounding variables will be the independent variables. Again, the necessity of using a random intercept and/or a random slope will be assessed using the -2 log likelihood test.

#### Economic evaluation

Cost-effectiveness analyses will be performed according to the intention-to-treat principle. Missing data will be imputed using Multivariate Imputation by Chained Equations (MICE) and pooled estimates will be calculated using Rubin’s rules [57]. Cost and effect differences will be estimated using linear mixed models or seemingly unrelated regression (SUR) analyses, depending on the number of required random intercepts and/or slopes [58]. Incremental cost-effectiveness ratios (ICERs) will be calculated by dividing the difference in costs by those in effects. BCa bootstrapping with 5000 replications will be used to estimate the uncertainty surrounding the ICERs and 95% confidence intervals around cost differences. Uncertainty will be shown by plotting cost-effectiveness planes and cost-effectiveness acceptability curves [59]. Sensitivity analyses will be performed to assess the robustness of the results (e.g. per-protocol analysis, complete-case analysis, human capital approach).

#### Process evaluation—mixed method analysis

Since both qualitative and quantitative data will be collected for the process evaluation, a mixed method analysis will be performed. Descriptive statistics (mean, SD, proportions) will be used to report the participants’ results of the closed questions and collected diary data. The open questions of the questionnaire will be listed, analysed and summarized. The main researcher (BW) and the implementation specialist (FvN) will code transcripts and other data qualitative obtained during the study. A framework analysis approach will be used to identify barriers and facilitators for adoption, implementation and maintenance [38]. Because both qualitative and quantitative data will be collected during the study, data triangulation will be used for the analysis. During this process, the researchers are mainly looking for outstanding differences or contrasts. Whether the data can support or supplement each other will also be looked into. To investigate the effect difference between different adherence groups within the intervention group, the group will be split in a low and high adherence group. A regression analysis will be performed to analyse the difference between these groups. Conclusions and claims shall be made carefully because of the between-study differences and explanation of heterogeneity [38]. To visualize the effect, a dose-response curve will be made.

### Interim analyses {21b}

Not applicable. Interim analyses will not be done in this study.

### Methods for additional analyses (e.g. subgroup analyses) {20b}

Depending on the course and outcomes of this study, additional sub-group analyses might be performed, e.g. between the different ASIA scores, how often someone has a PU, if someone has a PU during different times during the study, adherence to the intervention and the grade and location of the PU. The association between all risk factors and PU development/ incidence will also be looked at.

### Plans to give access to the full protocol, participant level-data and statistical code {31c}

Initially, the data will only be analysed for the purpose of this study. The data will then first become available for the involved researchers to identify other research purposes and kept for a maximum of 1 year. Subsequently, the research group intends to make the coded and de-identified syntaxes and data files accessible for further research and verification. However, access to the data set will be limited and access can only be allowed under certain conditions, because it will be hard to fully anonymize the dataset. If there are questions about the validity of the study results, the data may be made available to a requesting party with appropriate security measures. The party is only allowed to use the data to check the study results. The data of this study can only be reused for research in the same line as this study (similar goals, aims and purposes). Next to that, access to the data will only be granted when there is approval of the original research team and assurance that the receiving party will sufficiently protect the data. Extra safety precautions will be made when the data will leave the EU. The research group intend to share coded and de-identified syntaxes and data-files.

## Oversight and monitoring

### Composition of the coordinating centre and trial steering committee {5d}

This multicentre trial will be coordinated by researchers of the Vrije Universiteit Amsterdam together with researchers from the Amsterdam UMC and Reade. This trial will be directed by the principal investigator (TJ), coordinating investigator (SG) and by the executive researcher (BW). No overall steering committee will be involved in this study. All project members will play a part in the monitoring during the trial period and will meet weekly with a small group and every 3 months with all involved researchers.

### Composition of the data monitoring committee, its role and reporting structure {21a}

Because of the expected low risk of this study, a ‘Light Monitoring’ is chosen, hence without an additional monitoring committee. Since monitoring is outsourced in a lighter form, the task of monitoring will be exchanged with another study group that is independent during the monitoring or assessment.

### Adverse event reporting and harms {22}

All adverse events reported spontaneously by the participant or observed by the investigator or his staff will be recorded. The investigator will report all SAEs to the sponsor without undue delay after obtaining knowledge of the events. The sponsor will report the SAEs through the web portal toetsingonline.nl to the accredited METc that approved the protocol, in this case, Amsterdam UMC, location VUmc. The researcher will report the AE of SAE within the specified time as indicated. All AEs will be followed until they have abated, or until a stable situation has been reached. SAEs need to be reported until the end of the study within the Netherlands, as defined in the protocol.

### Frequency and plans for auditing trial conduct {23}

Once a year, the project group must submit a progress report to the Ethics Committee of Amsterdam UMC location VU and the funding agency (ZonMw). Auditing is the responsibility of the Ethics Committee who is allowed to randomly check WMO (Wet Medische-wetenschappelijk Onderzoek met mensen, i.e. Medical Research Involving Human Subjects Act) studies, however, this is not anticipated. If this trial is selected for an audit and omissions come to light, they are reported back to the principal investigator. A separate protocol and rules will be made to conform to the guidelines from the institute.

### Plans for communicating important protocol amendments to relevant parties (e.g. trial participants, ethical committees) {25}

All substantial amendments or important changes on the protocol will be indicated to the participating rehabilitation centres, sponsor and if needed to the METC and to the competent authority.

Non-substantial amendments will not be notified to the accredited METC and the competent authority, but will be recorded and filed by the sponsor. In addition, the journal, where the protocol paper and future articles were/will be published, will also be contacted.

### Dissemination plans {31a}

Implementation activities entail: understandable dissemination of results via rehabilitation specialists newsletters, patient information brochures and social media, presenting results at (inter-)national conferences, publication in high-impact peer-reviewed international journals and in Dwarslaesie Magazine (journal of the Dutch SCI patient organization) and the Nederlands Tijdschrift voor Revalidatiegeneeskunde (Dutch journal for rehabilitation medicine), and cost-effectiveness and potential scenarios for reimbursement discussions with health insurers. The participants are informed that after the research ends the sites Toetsingonline.nl and trialregister.nl will provide a summary of the results due to the registration of the study. Scientific publication or presentation by the Rehabilitation Center/Local Researcher, in whatever form, of the results of the study, will take place in accordance with the Protocol (publication agreements) and the indicated rules. All publications of the study will be coordinated by the Principal Investigator. The main researchers have the first right to publish the results of the project in scientific journals, dissertations from other academic media and will always consult the involved rehabilitation centre and/or the local investigator about a proposed publication. The final results will be submitted to a peer-reviewed scientific journal within the first year after completing the trial.

## Discussion

The aim is to conduct this study in all eight Dutch rehabilitation centres with a specialized SCI unit with motivated rehabilitation specialized and to recruit individuals with a SCI throughout the Netherlands. In addition to the complexity of setting up a good research infrastructure, this will be the first study in the field of ES for PU prevention with this relatively large number of participants (*N* = 100).

This trial will provide information about the (cost-)effectiveness of daily ES to prevent recurrent PUs and the influence of ES on the PU risk factors compared to usual care. This information is important because PUs are still a major problem within individuals with a SCI. The mechanisms by which ES can improve PU risk factors and daily living also need to be better understood. In this study, the control intervention is usual care (standard care) in the home situation, which will not be controlled in any way to make the study results as generalizable to daily practice as possible so that the effect of adding ES of the hamstrings and gluteal muscle can be identified in a real-world setting. If this study shows a positive effect, it could lead to a wide implementation of electrical stimulation in both rehabilitation centres and in people's home rehabilitation process. That is why the researchers have chosen to perform a problem analysis study to implement the results as well as possible, taking into account barriers and facilitators

## Trial status

The study is expected to be open to enrolment and start active recruiting in May 2021 with the anticipated completion date of June 2023. The total duration of the study is from May 2021 till June 2023. The trial is registered in: NTR, ID: NL9469, Protocol version 2.5 dated 20-04-2021.

## Supplementary Information

**Additional file 1:** **Appendix 1.** RE-AIM model.

**Additional file 2:** **Appendix 2.** Participant informed consent.

**Additional file 3:** **Appendix 3.** Data Management Plan (SCI PREVOLT) version 1.

## Abbreviations

PU(s): Pressure ulcer(s)
SCI: Spinal cord injury
ES: Electrical stimulation
DON: Dwarslaesie Organisatie Nederland
FSS: Fatigue Severity Scale
HADS: Hospital Anxiety and Depression Scale
PASIPD: Physical Activity Scale for Individuals with Physical Disabilities
PSQI: Pittsburgh Sleep Quality Index
SIP-68: 68-item Sickness Impact Profile
USER-P: Utrecht Scale for Evaluation of Rehabilitation-Participation (USER-participation)
WHOQOL-5: World Health Organization Quality of Life-5
(S)AE: (Serious) adverse event
METC: Medical research ethics committee (MREC); in Dutch: medisch-ethische toetsingscommissie (METc)
WMO: Medical Research Involving Human Subjects Act; in Dutch: Wet Medisch-wetenschappelijk Onderzoek met Mensen

## Footnotes

1 In this study, a chronic SCI is defined as ‘Every individual with an SCI who has been discharged from a rehabilitation center.’

2 Potential participants who underwent flap surgery or some other form of plastic surgery can still be included if the stimulation can induce a good muscle contraction.

3 According to the European pressure Ulcer Advisory Panel [22].
