# Supplementary material for: Electrical stimulation to prevent recurring pressure ulcers in individuals with a spinal cord injury compared to usual care: the Spinal Cord Injury PREssure VOLTage (SCI PREVOLT) study protocol
Source: Trials. 2022 Feb 16;23:156. doi: 10.1186/s13063-022-06088-0 (PMC8848924; doi:10.1186/s13063-022-06088-0)
Supplement: Supplementary file 1 — Additional file 1: Appendix 1. RE-AIM model. [file 13063_2022_6088_MOESM1_ESM.pdf]

Appendix 1 RE-AIM model:

Objectives that relate to the different dimensions of the RE-AIM model (52):

Reach

- The characteristics of the group of participant based on background, demographic characteristics, age, gender, duration of SCI, level of SCI and how many PUs in the past
- The characteristics of the care takers that were involved in the implementation
- The characteristics of the rehabilitation centers that were attracted to participate

Effectiveness

- Difference in effectiveness of the surface electrodes versus the lycra garment on participants pressure ulcer incidence or recurrences, secondary outcomes and variation in results between locations at 3 months, 6 months and 12 months after baseline.
- Dose-response association between degree of adherence and effectiveness

Adoption

- Sources and procedures for recruitment of centers to participate in the intervention and difference between the rehabilitation centers and their willingness to adopt the intervention
- Perceived barriers and facilitators for the rehabilitation centers for the adoption of the intervention, what worked well and less well in terms of recruitment

Implementation

- The extent to which the participants adhered the intervention, and difference between subgroups who did or did not complete (fidelity)
- Participants views and experiences of the intervention, which elements were viewed as helpful and unhelpful and their perceived effectiveness
- Care takers views and experiences of the intervention, which elements were viewed as helpful and unhelpful and their perceived effectiveness
- Rehabilitations centers views and experiences of the intervention, which elements were viewed as helpful and unhelpful and their perceived effectiveness
- Perceived barriers and facilitators for the implementation of the ES for rehabilitation centers, the care takers and participants

Maintenance

- Perceived barriers and facilitators for sustained implementation of the treatment for a rehabilitation specialist or rehabilitation center
- Recommendations for improvement of the intervention beyond the research setting; what are likely modifications or adaptations that will need to be made to sustain the initiative over time (like lower the costs, employers, intensity, settings, intervention method)

Interviews

After 3 months (T1) and after 12 months (T3) a select sample of participants, caretakers and specialist will be asked to participate in individual interviews. In total 6-10 interviews will be conducted on each moment. The researchers will invite purposely individuals, to retrieve a broad variation in differences in level of adherence, satisfaction, age, gender and ulcer development. The purpose of these interviews are:

- To find out barriers of the current intervention.
- Hearing ideas for improvement of the intervention (garments/stimulator/cycle).
- How they see the future of ES.
- How ES can be a bigger part of rehabilitation.
- Explore suggestions for future implementation.

Next to this, interviews will be conducted with important broader stakeholders like therapists, physician assistants, rehabilitation specialist, redaction, health insurers and guideline producers, not taking part in the study. This will be done parallel to the intervention study. This is important to improve and ensure a future implementation of ES. These interviews will be done during the measurement period but mostly between the T2 and T3 measurements. During these interviews potential facilitators and barriers for implementation beyond the research setting will be identified.

Interviews will be recorded with an Olympus WS-853 which can only collect audio so there will not be any collection of imaging or video. The Olympus WS-853 will give MP4 files, between the 25 and 200 mb per interview. These files will be stored encrypted in Research Drive in a folder with restricted access. If the transcribing will be outsourced to a company (for example TiptopGlobal) a joint processor agreement will be made in consultation with IXA or privacy lawyers form the VU. The raw interviews will be exchanged with surffilesender protected with a password. Subsequently, the transcribed interviews will be stored on a research drive for further elaboration. The raw interviews will be removed. There is already made a joint

processor agreement between the VU, Reade and VUmc so the data can be exchanged between these companies.

Groups consultation/Focus groups

Around the last measurements (T3), a group meeting with import stakeholders for implementation will be set-up. This group composition will exist of: rehabilitation physicians, physician assistants, physiotherapists, nurse practitioners, coordinators, managers, research assistants and other types of researchers. These people either had an important role during the study or are familiar with the research, but did not contribute. The purpose of this meeting is to get an idea of how people envision future implementation, the network infrastructure around ES and the future of ES in rehabilitation. The end goal of this meeting is to make a plan for implementation, the infrastructure and future network. The plan and ideas that emerge during this meeting will be processed/included in the implementation plan.

Diary data of participants

Participants in the intervention group will be asked to keep a diary of usage. The overall usage will be used to answer questions about adherence. These answers have an important role in the dimensions ‘effectiveness’ and ‘implementation’.

Questionnaire data of participants

Within the self-administrated questionnaire about the usability of the electrical stimulation system questions from the RE-AIM model are included. Especially the open questions will play an important role in the mixed method analysis.

Field notes

The main researchers will be asked to make field notes of all relevant information and interesting events. This will be done throughout the different theme’s and aspect of the RE-AIM model.

Schedule of Assessment

	T0				T1				T2				T3			
**Measurement**	**P**	**CT**	**RB**	**SH**	**P**	**CT**	**RB**	**SH**	**P**	**CT**	**RB**	**SH**	**P**	**CT**	**RB**	**SH**
**Interviews:** ***Category Adoption***																
Of the centers					X	X	X						X	X	X	
Participants intervention					X	X	X						X	X	X	
Barriers					X	X	X						X	X	X	
Future logistics					X	X	X					X	X	X	X	X
Interviews: Category Implementation																
Barriers					X	X	X					X	X	X	X	
Participant and daily life					X	X	X						X	X	X	
Interviews: Category Maintenance																
Barriers					X	X	X					X	X	X	X	
Maintaining ES					X	X	X						X	X	X	
Future maintenance					X	X	X					X	X	X	X	X
**Questionnaire:** ***Category Adoption***																
Participants intervention	X				X				X				X			
**Questionnaire:** ***Category Implementation***																
ES in daily life	X				X				X				X			
**Questionnaire:** ***Category Maintenance***																
Maintaining ES	X				X				X				X			
Future maintenance	X				X				X				X			
**Diary*****: Category Reach***																
Adaptation to ES					X				X				X			
**Diary*****: Category effectiveness***																
Effect and results					X				X				X			
**Diary*****: Category adoption***																
Participants intervention					X				X				X			
**Diary*****: Category maintenance***																
Maintaining ES					X				X				X			
**Focus Group:** ***Category adoption***																
Future logistics													X	X	X	X
**Focus Group:** ***Category Implementation:***																
Future implementation													X	X	X	X
**Focus Group:** ***Category maintenance***																
Future maintenance													X	X	X	X

Table 2: P = Participants, **CT** = care takers, **RB** = rehabilitation specialist, **SH** = stakeholders
