# Supplementary material for: Electrical stimulation to prevent recurring pressure ulcers in individuals with a spinal cord injury compared to usual care: the Spinal Cord Injury PREssure VOLTage (SCI PREVOLT) study protocol
Source: Trials. 2022 Feb 16;23:156. doi: 10.1186/s13063-022-06088-0 (PMC8848924; doi:10.1186/s13063-022-06088-0)
Supplement: Supplementary file 2 — Additional file 2: Appendix 2. Participant informed consent. [file 13063_2022_6088_MOESM2_ESM.pdf]

Appendix 2: **Participant informed consent (translated to English):**

SCI PREVOLT: Spinal Cord Injury PREssure VOLTage

- I have read the information letter and I have been given the opportunity to ask questions about the study. My questions have been answered sufficiently. I had enough time to decide whether I wanted to participate in the trial or not.
- I know that participation in this study is voluntary. I also know that I can decide to stop participating in this trial at any time without giving an explanation.
- I know that this concerns a study performed by the VU Amsterdam in collaboration with Reade and Amsterdam UMC, location VUmc. This means that my data are exchanged and used by those different parties. I give permission for this.
- I give permission to inform my general practitioner/specialist about my participation to this trial.
- I give permission for the collection and use of my data. The measurement procedures were explained to me in the information letter and appendix. My data are used to answer the research questions of this study, which were explained to me in the information letter.
- I know that some people can get access to all my data for auditing purposes. These people were listed in the information letter. I give permission to these people to access my data if necessary.
- I know that in case of doubt about the study results, data can be made available to a party with appropriate safety measures. This party may only use the data to verify the study results.
- I know that as a woman cannot get pregnant during the study.
- I know that my data will be stored for 15 years after the end of this study and that the details about this were provided in the information letter.
- I know that as soon as I agree to participate to this study, I give permission to inform my general practitioner and/or treating specialist to be informed if unexpected findings or additional findings that are (or may be) important for my health are found.
- I …. give □ **do**
  □ **do not**
  permission to share my data with other parties for future research (with similar research purposes). I know that my data will be stored for this for 15 years.
- I …. give □ **do**
  □ **do not**
  permission to contact me after this study to ask whether I want to participate in a (possible) follow-up study
- I want to participate in this research

Name participant: ________________________________

Signature: Date : __ / __ / __

____

I declare that I have fully informed this participant about the study mentioned above. If information becomes known during the study that could influence the participants consent, I will inform him/her in on time.

Name researcher (or his representative): ________________________________

Signature: Date : __ / __ / __

____
