# Supplementary material for: Electrical stimulation to prevent recurring pressure ulcers in individuals with a spinal cord injury compared to usual care: the Spinal Cord Injury PREssure VOLTage (SCI PREVOLT) study protocol
Source: Trials. 2022 Feb 16;23:156. doi: 10.1186/s13063-022-06088-0 (PMC8848924; doi:10.1186/s13063-022-06088-0)
Supplement: Supplementary file 3 — Additional file 3: Appendix 3. Data Management Plan (SCI PREVOLT) version 1. [file 13063_2022_6088_MOESM3_ESM.pdf]

Daily electrical stimulation to prevent recurring pressure ulcers in patients with a spinal cord injury compared to usual care

1. General features of the project and data collection

1.1 Project leader contact details

Prof. dr. Thomas W.J. Janssen
Faculty of Behavioural and Movement Sciences, Dept. Human Movement Sciences
Room MF-D625, Van der Boechortstraat 7, 1081 BT Amsterdam
T +31 20 59 88586 | M: +31 6 46225856
t.w.j2.janssen@vu.nl

Reade, Rehabilitation & Rheumatology
Overtoom 283
1054 HW Amsterdam
+31206071699
t.janssen@reade.nl

1.2 I have composed my DMP with the assistance of a data stewardship (or management) expert. List his or her name, function, organisation/department, phone number and email address.

- The expert is connected to my department or institution (please explain his/hr expertise related to data stewardship)

Jessica Hrudey: research.data.fgb@vu.nl / e.j.hrudey@vu.nl
telefoonnummer: (020)5989662

1.3 In collecting data for my project, I will do the following:

- Generate new data

Data will be gathered during the trial using online questionnaires, physical measurements, interviews and during a focus group (participants will provide informed consent). All the participant data will be coded and de-identified to protect the privacy as good as possible or if possible anonymized /pseudonymized. So it can be published through open access and on the basis of the FAIR principles.

1.4 In my research, I will use:

- A combination of quantitative and qualitative data

1.5 I will be reusing or combining existing data, and I have the owner's permission for that.

- Yes, I have permission to use the data

1.6 In collecting new data, I will be collaborating with other parties.

- Yes, the new data will be (partly) provided by a project partner or supplier
- Yes, we have reached agreements on the user rights of the data used in the project
- Yes, I will collect the new data in conjunction with other researchers or research groups

The project is a multicenter trial, consisting of 7 rehabilitation centers in the Netherland. A research agreement will be developed for the participating centers.

1.7 I am a member of a consortium of 2 or more partners. Clear arrangements have been made regarding data management and intellectual property. (also consider the possible effect of changes within the consortium on issues of data management and intellectual property)

- Yes, clear arrangements have been made regarding data management and intellectual property through a consortium agreement

1.8 I can give an estimate of the size of the data collection; specifically, the number of participants or subjects ("n=") in the collection and its size in GB/TB

- Yes (please specify)

Number of expected subjects= 100

I anticipate the size of the rawdata not to be greater than **1GB**, as we intend to only publish our raw datasets and data documentation.

The data collection will be done and saved on different databases.

For the interviews, the audiofiles (MP3) will be collected with the olympus WS-853. This will take approximately up to **38 GB** uncompressed WAV documents and will be saved on a local drive of the VUMC, with restricted access. This will also be saved on Research drive.

When these interviews are transcribed they will be transferred to the VU and this will take between 25-200mb per file. The expectation is that it will take up to **10,2 GB** in total.

For the questionnaires and diaries, we expect to use up to **1,6 Gb** in total when exported

as an PDF.

The pictures of the sacral area will take about **37 GB** at maximum. This data will be saved on a local drive of Reade and on Research Drive, both with restricted access.

For the measurements on-site, like the Bloodflow, Muscle thickness and pressure distribution we expect to need at least **13 Gb** of space. This data will be saved locally at VU one drive before being uploaded to Research Drive.

Total space usage in the VUMC would be around: 48,2 GB

Total space usage in Reade would be around: 51,6 Gb

Total space usage in the VU would be around: 63,8 GB

Total space usage in Research Drive would be expected to be around: 105 Gb

1.9 The following end products I will make available for further research and verification (please elaborate briefly)

- Syntaxes
- Documentation of the research process, including documentation of all participants
- Audiovisual material/ Images
- Raw data

Qualitative data will be gathered using physical measurement and e.g. Qualtrics, SnapSurvey or Survalizer. This data will be analyzed and will be analyzed using SPSS.

Quantitative data will be gathered using an online questionnaire which will be reported as descriptive statistics (mean, SD, proportions). Also, there will be gathered data with recoding devices that will be transcribed in (F4, Express-scribe, otranscribe or as seems fit during the process) and analyzed on barriers and facilitators with the Fleuren framework.

1.10 During the project, I will have access to sufficient storage capacity and sites, and a backup of my data will be available. (please elaborate briefly)

- Yes, I will make use of my institution's standard facilities for storage and backup of my data

For storing the data, the storage facilities of the VU Amsterdam will be used, as well as the storage facilities of participating rehabilitation centers Reade and of the VUMC. There will be made a joint controller agreement between these companies.

The main storage will be done on Research-drive from the VU. But also in Reade and VUMC a local secured drive with limited access will be used.

2. Legislation (including privacy)

2.1 I will be doing research involving human subjects, and I am aware of and compliant with laws and regulations concerning privacy sensitive data.

- The Wet Medisch-Wetenschappelijk Onderzoek met Mensen (WMO, or Medical Research (Human Subjects) Act) applies to my project; I will have it reviewed by a Medical Research Ethics Committee. In addition I will comply with the Kwaliteitsborging Mensgebonden Onderzoek (Quality Assurance for Research Involving Human Subjects)
- Yes, I will involve human subjects in my research. I will comply with the Algemene Verordening Gegevensbescherming (AVG)

2.2 I will be doing research involving human subjects, and I have (a form of) informed consent from the participants for collecting their data.

- Yes, and this informed consent allows for the reuse of data (note that in the Code of Conduct for Medical Research, ‘reuse’ is also referred to as ‘further use’)

2.3 I will be doing research involving human subjects, and I will protect my data against misuse.

- Yes, the data will be pseudonymised. (please explain how this will be done, and by which organisation) and

Participants sensitive data will be coded and de-identified to protect the privacy as good as possible or if possible pseudonymized.

A case record form ((CRF); participant data file) will be made where each participant will get a participant number existing of two letters and two numbers. These two letters refer to the rehabilitation center they come from and the two numbers will be their inclusion number (1-15 for each center). Personal information will be saved in the participants delegation log, like: name, email, phone number, address, etc. The key to the code will be safeguarded by the principal investigator, coordinating investigator and the executive investigator. A document will be created within this file to record who has been granted access or has had access during the course of the project. The CRF file and the scanned informed consents will be kept on a separate drive of Research drive or the VU where the rights of access will be strictly regulated. The paper consent will be kept in a locked cabinet inside the VU, Reade or other participating rehabilitation centers. The room and closed will be locked overnight and there will be restricted access to this room.

The direct audiofiles will be transcribed and pseudonymized as good as possible. All the other quantitative data will be de-identified and if needed there can be chosen to generalize the extreme values.

In accordance with the VSNU Code of Conduct for Research Integrity and VU/FGB RDM Policies, all data collected will be kept and stored for at least 15 years after the last treatment is given and at least 10 years after the last article is published. Data that are stored longer than that will depend on what participants consented to. The participants will be informed in writing about these data storing and handling procedures. The handling of personal data and other data will be in line with the GDPR (in Dutch: AVG). We anticipate to use DarkStor as archive where the data will be stored for a period of 15 years. DarkStor is the most suitable candidate because of the longterm storage of

privacy-sensitive data.

2.4 I will stick to the privacy regulations of my organisation

- Yes

3. Making data findable

3.1 The data collection of my project will be findable for subsequent research. E.g., on a catalogue, a web portal, or through the search enginge of the repository (note: this is key item 3, which you should report to ZonMw at the end of your project).

- No, I have not yet chosen an archive or catalogue/web portal

It is both VU and FGB policy that all data are archived upon completion of research (or when a research article is published) it needs to be registered in PURE.

The data collection will not be stored in PURE but there will be a description of the data that is registered and where to find it.

3.2 I will use a metadata scheme for the description of my data collection (note: this is key item 7, which you should report to ZonMw at the end of your project).

- Yes, I will use a generic metadata scheme (please specify)

Because of the registration in PURE, we will automatically make use of the CERIF metadata scheme.

3.3 I will be using a persistent identifier as a permanent link to my data collection (note: this is key item 1, which you should report to ZonMw at the end of your project).

- Yes, in addition to the DOI code I will be using another persistent identifier (please specify)

I will get a persistent identifier while storing the data in DarkStor.

During the course of the study there can be chosen to register all the research activities in OSF.

4. Making data accessible

4.1 Once the project has ended, my data will be accessible for further research and verification.

- Yes, after an embargo period (please explain)

Data will first be analyzed by the researchers involved in the project. After this, the data will be held (up to a year) to look for other research purposes within the participating centers. After this the data will be made accessible for further research and verification.

4.2 Once the project has ended, my data collection will be publicly accessible, without any restrictions (open access).

- No, there will be access restrictions to my data collection (please explain)

There will be restricted access to the data collection and access can be permitted under certain conditions. This is due to the fact that the data will be hard to fully anonymize.

If there are questions about the validity of the study results the data may be made available to a requesting party with appropriate security measures. The party is only allowed to use the data to check the study results.

The data of this study can only be reused for research in the same line as this study (same kind of goals, aims and purposes). Next to that, there will only be granted access to the data when there is approval from the original research team and assurance that the receiving party will sufficiently protect the data. There will be made extra safety precautions when the data will leave the EU.

We intend to share coded and de-identified syntaxes and datafiles.

4.3 I have a set of terms of use available to me, which I will use to define the requirements of access to my data collection once the project has ended (please provide a link or persistent identifier; also note that this is a key item 4, which you should report to ZonMw at the conclusion of your project).

- Not yet, my institution will draft a set of terms of use with the help of a legal advisor

4.4 In the terms of use restricting access to my data, I have included at least the following:

- The manner in which the data set can be accessed

- Collaboration in using the data set, including agreements on publication and authorship
- Whether or not the data set may be linked with another data set (for reasons of privacy)
- The approval of the participants allows for further research using this data set
- Agreements on methodology
- A steering committee, programme committee or project leader will be charged with approving data requests
- The permitted period of use of the data set

The exact terms of use will be determined during the project. These terms will be approved by lawyers of IXA and the privacy lawyers at the VU.

5. Making data interoperable

5.1 I will select a data format, which will allow other researchers and their computers (machine actionable) to read my data collection (note: this is key item 5, which you should report to ZonMw at the end of your project).

- Yes (please specify)

The structured quantitative raw data will be saved as a .csv format (open format copy) so it can be accessed on multiple software programs. After this, most of the data will be analyzed with SPSS but there will be tried to save it in an open format.

Quantitative data will be made available as SPSS, STATA, MLWIN or R files (depending on the program that was used for the
respective analyses). Syntaxed will be made available as SPSS, STATA, MLWIN or R files (depending on the program that was used for the respective analyses). All codes used will making syntaxes or scripts will be saved in a proprietary format like txt.

Qualitative data will be transcribed with one of the follow software programs: F4, Express-scribe or otranscribe. When data will be transcribed by an independent company a software program will be chosen as seems fit. Analyses of the qualitative data will be done with ATLAS.ti.

Other study documentation will be made available as PDFs, .txt. .csv file.
Transcribed interview scripts will be made available as PDFs, .txt. .csv file. How both will be made available will be determined as seem fit, at the time.

5.2 I will select a terminology for recording my data (e.g., code, classification, ontology) that allows my dataset to be linked or integrated with other datasets (note: this is key item 6, which you should report to ZonMw at the conclusion of your project).

- Yes, metadata standard (please specify)

The metadata standard of CERIF will be used.

Terminology that will be used are:

- Daily Electrical Stimulation

- Electrical stimulation (ES)

- Pressure ulcer (PU)/ Pressure ulcers (PU's) and Decubitus

- Spinal cord injury (SCI)

- Asia impairment scale: describes a person's functional impairment as a result of an SCI (sensory- and motor function)

- Interface pressure distribution: The overall sitting pressure between soft tissue (muscle) and bony prominence (sacrum or ischial tuberosity's region)

- Healing tendency: How fast a wound heals.

5.3 I will be doing research involving human subjects, and I have taken into account the reuse of data and the potential combination with other data sets when taking privacy protection measurements.

- Yes, the participants have given their permission for reuse of the data, and the data have been pseudonymised

The data will be extensively de-identified and pseudonymized as good as possible. One of the conditions (terms) for reuse is that reusers do not try to actively identify participants. Prior to making the data available the researchers will make sure to reduce the risk to identify an individual. This will be done by taking steps and deleting, de-identified, generalizing extreme values to make sure there is a low risk by sharing while still being useful.

6. Making data reusable

6.1 I will ensure that the data and their documentation will be of sufficient quality to allow other researchers to interpret and reuse them (in a replication package).

- I will document the software used in the course of the project (please specify)
- I will document the research process (please explain)
- I will perform quality checks on the data to ensure that they are complete, correct and consistent (please explain)

Data will be stored and managed in accordance with the quality standards of the VU.

All data collections, changes and interpretation will be documented in codebooks or txt. files. This will be done to make a review and reuse easier.

All the data from the questionnaire, diary, interviews en physical measurement will be copied and saved. So that it can still be checked or read in the future.

Data that will be entered into a database will either be entered into a database such as Access or FileMakerPro via a data entry screen. Questionnaire data can probably be exported directly in SPSS where it will be manually checked and cleaned. If this is not possible, the data will be manually entered into SPSS.

Every step in data cleaning in the scripts/syntaxes will be documented and recorded in a logbook. Each cleaning step will be discussed within the involved research team so that everyone is doing it consistently and persistent. When students get involved in data collection/cleaning there will be made a short protocol or steps that need to be taken these will be readable and easy to follow for everyone.

6.2 I have a number of selection criteria, which will allow me to determine which part of the data should be preserved once the project has ended. (see also question 1.9 and 6.1)

- No

In accordance with the VSNU Code of Conduct for Research Integrity and VU/FGB RDM Policies, all data collected will be kept and stored for at least 10 years after the last research article is published and/or at least 15 years after the last treatment is given (whichever is longer). Data that are stored longer than that will depend on what participants consented to.

6.3 Once the project has ended and the data have been selected, I can make an estimate of the size of the data collection (in GB/TB) to be preserved for long-term storage or archival.

- Yes (please specify)

The points below are the same as in point 1.8

'I anticipate the size of the rawdata not to be greater than ***1GB****, as we intend to only publish our raw datasets and data documentation.*

The data collection will be done and saved on different databases.

For the interviews, the audiofiles (MP3) will be collected with the olympus WS-853. This will take approximately up to ***38 GB*** *uncompressed WAV documents and will be saved on a local drive of the VUMC, with restricted access. This will also be saved on Research drive.*

When these interviews are transcribed they will be transferred to the VU and this will take between 25-200mb per file. The expectation is that it will take up to ***10,2 GB*** *in total.*

For the questionnaires and diaries, we expect to use up to ***1,6 Gb*** *in total when exported as an PDF.*

The pictures of the sacral area will take about ***37 GB*** *at maximum. This data will be saved on a local drive of Reade, with restricted access.*

For the measurements on-site, like the Bloodflow, Muscle thickness and pressure distribution we expect to need at least ***13 Gb*** *of space. Whether this data will be saved at the VU directly or in Reade is not yet known and will be determined later in the process.*

Total space usage in the VUMC would be around: 48,2 GB

Total space usage in Reade would be around: 51,6 Gb

Total space usage in the VU would be around: 63,8 GB

The main data will be archived at the VU, probably on DarkStore or something similar because of the privacy-sensitive data. This includes the raw datasets, the exported questionnaires, diaries and the transcribed interviews. This probably will take about 25,8

GB
Where the audio files and photos will be archived, still needs to be determined by the research group.

6.4 I will select an archive or repository for (certified) long-term archiving of my data collection once the project has ended. (note: this is a key item, which you should report to ZonMw at the conclusion of your project)

- Yes, and this archive has a data seal of approval (please specify the archive)
- Yes, and this archive meets certification criteria and intends to get certified (please explain how your data will remain accessible and reusable in the long term)

The final choice about the repository will be made during the course of the study. We anticipate using Darkstore because of the agreement with the VU.

6.5 Once the project has ended, I will ensure that all data, software codes and research materials, published or unpublished, are managed and securely stored. Please specify the period of storage.

- Yes, in accordance with VNSU guidelines (please specify the number of years)

We anticipate to use DarkStor where the data will be stored for a period of 15 years. DarkStor is the most suitable candidate because of the longterm storage of privacy-sensitive data.

6.6 Data management costs during the project and preparations for archival can be included in the project budget. These costs are:

- Unknown (please explain)

Not all costs of data management are yet known.

For transcribing the qualitative data: there can be chosen to let this be done by students or to let an independent company transcribe the interviews (costs are not yet determined).

Other data management costs are not yet determined in the project budget.

The cost of archiving in DarkStore will be 30 euro's for 15 year. This is for <50Gb and for >50Gb storage.

6.7 The costs of archiving the data set once the project has ended are covered.

- Not yet (please explain)

Costs for data archiving will be paid by the VU up to 50 GB. If the data collection will be

expected more than 50 GB it needs to be approved by the faculty.
